# Contact force sensing in ablation of ventricular arrhythmias using a 56-hole open-irrigation catheter: a propensity-matched analysis

**DOI:** 10.1007/s10840-020-00756-4

**Published:** 2020-05-21

**Authors:** Ahmed I. Elbatran, Anthony Li, Mark M. Gallagher, Riyaz Kaba, Mark Norman, Elijah R. Behr, Manav Sohal, Abhay Bajpai, Zia Zuberi, Magdi M. Saba

**Affiliations:** 1 Cardiology Clinical Academic Group, St. George’s University Hospitals NHS Foundation Trust, St. George’s, University of London, London, UK; 2 Department of Cardiology, Ain Shams University, Cairo, Egypt

**Keywords:** Ablation, Complications, Contact force, Irrigated radiofrequency, Ventricular tachycardia, Ventricular ectopy

## Abstract

**Purpose:**

The effect of adding contact force (CF) sensing to 56-hole tip irrigation in ventricular arrhythmia (VA) ablation has not been previously studied. We aimed to compare outcomes with and without CF sensing in VA ablation using a 56-hole radiofrequency (RF) catheter.

**Methods:**

A total of 164 patients who underwent first-time VA ablation using Thermocool SmartTouch Surround Flow (TC-STSF) catheter (Biosense-Webster, Diamond Bar, CA, USA) were propensity-matched in a 1:1 fashion to 164 patients who had first-time ablation using Thermocool Surround Flow (TC-SF) catheter. Patients were matched for age, gender, cardiac aetiology, ejection fraction and approach. Acute success, complications and long-term follow-up were compared.

**Results:**

There was no difference between procedures utilising either TC-SF or TC-STSF in acute success (TC-SF: 134/164 (82%), TC-STSF: 141/164 (86%), *p* = 0.3), complications (TC-SF: 11/164 (6.7%), TC-STSF: 11/164 (6.7%), *p* = 1.0) or VA-free survival (TC-SF: mean arrhythmia-free survival time = 5.9 years, 95% CI = 5.4–6.4, TC-STSF: mean = 3.2 years, 95% CI = 3–3.5, log-rank *p* = 0.74). Fluoroscopy time was longer in normal hearts with TC-SF (19 min, IQR: 14–30) than TC-STSF (14 min, IQR: 8–25; *p* = 0.04).

**Conclusion:**

Both TC-SF and TC-STSF catheters are safe and effective in treating VAs. The use of CF sensing catheters did not improve safety or acute and long-term outcomes, but reduced fluoroscopy time in normal heart VA.

## Introduction

Catheter ablation is an important treatment for ventricular arrhythmias (VA) [1], but long-term outcomes remain suboptimal. Measuring the contact force (CF) between the radiofrequency (RF) ablation catheter and myocardium aims to improve mapping accuracy, energy delivery and procedure safety, by optimising catheter-tissue contact. In comparison to the large volume of data available on measuring CF in AF ablation, much less work has focused on its real-world usefulness in VA.

This study compares acute success, complication rate and long-term arrhythmia-free survival in catheter ablation procedures for VA using the 56-hole RF ablation Thermocool Surround Flow (TC-SF) catheter versus the Thermocool SmartTouch Surround Flow (TC-STSF) catheter (both Biosense Webster, Diamond Bar, CA, USA). The latter combines the ability to monitor the magnitude and direction of catheter-tissue contact with entire tip irrigation.

## Methods

### Patient population

**Fig. 1:**
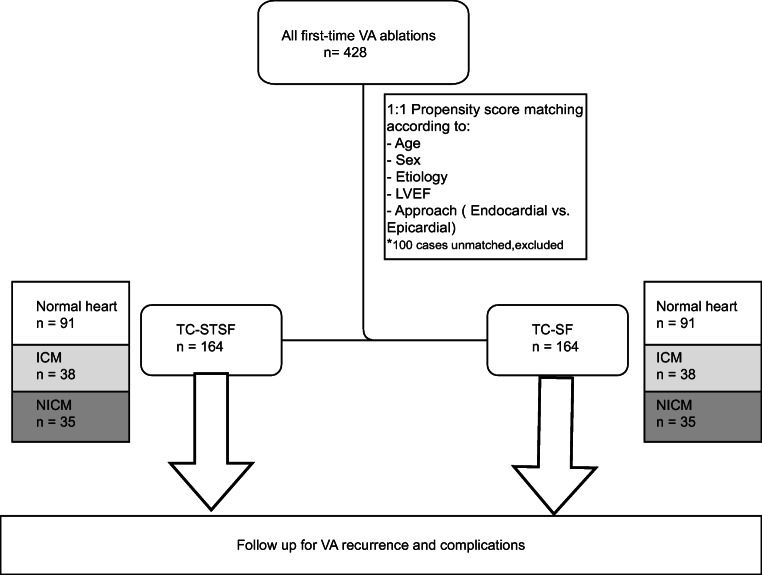
Flowchart for the study design. ICM = ischemic cardiomyopathy; LVEF = left ventricular ejection fraction; NICM = non-ischemic cardiomyopathy; TC-SF = Thermocool Surround Flow; TC-STSF = Thermocool SmartTouch Surround Flow; VA = ventricular arrhythmia

Procedure- and outcome-related data were collected for the 164 patients undergoing first-time ablation for VA using TC-STSF catheter between 2016 and 2018 at St George’s University Hospital, London, UK (91 with structurally normal heart, 38 with ischemic cardiomyopathy (ICM) and 35 with non-ischemic cardiomyopathy (NICM)). From a database of 264 first-time VA ablation procedures performed in the same institution using TC- SF catheter, one procedure was propensity-matched against each TC-STSF procedure (Fig. 1). The study was approved by the local institutional research ethics committee.

There were 209 procedures for ventricular ectopy (VE) and 119 for ventricular tachycardia (VT). The data comprised baseline patient demographics, left ventricular ejection fraction (LVEF), presence and indication for intracardiac devices, number of prior ablations and underlying cardiac aetiology. All patients underwent a transthoracic echocardiogram (TTE) or cardiac magnetic resonance imaging (cMRI), or both, pre-procedurally. Coronary angiography was performed in all cases of structural heart disease and was discretional in the case of normal heart VAs.

The indication for VA ablation was categorised as symptomatic despite medical therapy, presumed VA-induced cardiomyopathy unresponsive to medical therapy, recurrent implantable cardioverter defibrillator (ICD) shocks or VT storm. Antiarrhythmic drug therapy was discontinued for 5 half-lives, except for patients presenting with VT storm or those on Amiodarone. Scar-dependent VT ablation was performed under general anaesthesia or conscious sedation. Sub-xiphoid epicardial approach was undertaken at the discretion of the operator and was based on clinical aetiology, pre-procedural imaging or prior procedural mapping.

### Definitions

VA was defined as either VT or VE. Normal heart VA was defined as the absence of structural abnormality on TTE or cMRI. Acute procedural success was defined as non-inducibility of all VT(s) and the complete elimination of scar-related abnormal electrograms in VT cases and as > 80% reduction in clinical VE frequency despite isoprenaline challenge, during a 30-min wait period in VE cases. VA recurrence was defined as sustained monomorphic VT (> 30 s) or VT requiring ICD therapy (anti-tachycardia pacing or shock) for patients undergoing VT ablation and as < 80% reduction of the burden of clinical ectopy on 24-h Holter monitoring in patients undergoing VE ablation.

### Ablation strategy

Dual left ventricular (LV) access was employed in scar-dependent VT cases, using both the trans-septal and the retrograde aortic approaches. Anticoagulation during LV procedures was maintained with unfractionated Heparin and titrated to achieve an activated clotting time of 300–400 s.

Patients underwent electroanatomical mapping during VA or in sinus rhythm with either the CARTO 3 (Biosense-Webster, Diamond Bar, CA, USA) or the Ensite Velocity/Precision System (St. Jude Medical, St Paul, MN, USA) and TC-SF or the CARTO 3 system and TC-STSF (Biosense-Webster, Inc.), with TC-STSF largely supplanting TC-SF in 2016. Both catheters have a 3.5 mm tip with 56 pores in the distal electrode through which normal saline was infused at a rate of 2 ml/min during mapping. While using the TC-STSF catheter, the average and maximum CF between the catheter tip and the myocardium were measured and expressed in grams (g), aiming for CF of 10–40 g. Mapping was supplemented by multi-electrode mapping catheters, which were more commonly used in the latter years of the study.

Voltage mapping was performed during sinus rhythm or right ventricular (RV) pacing at the outset. Programmed stimulation was performed with up to 3 extrastimuli at 2 cycle lengths. Ablation was performed in the power control mode, with temperature cut-off of 40 °C, maximum power of 40 W (rarely up to 50 W on the interventricular septum), flow rate of 17 ml/min, duration of RF application of 60 s at each point, with lower energy and shorter duration at aortic sinus of Valsalva and para-Hisian locations. Ablation was terminated immediately if there was an impedance rise or an audible steam pop.

For VT cases, ablation of all clinical VTs was attempted. Abnormal scar-related electrograms were targeted with focal ablation in scar with endocardial voltage < 1.5 mV, aiming at the elimination of near-field electrograms, impedance drop of 10 Ω and loss of capture with moderate output pacing [2].

For VE cases, ablation was performed at the earliest site of local activation and/or the site of best pacemap (> 90%) match to the clinical VE.

### Follow-up

All major intra- and post-procedural complications were recorded up to 30 days. Antithrombotic therapy was recommended for 1–3 months after LV endocardial procedures with either direct oral anticoagulants or warfarin with a goal international normalised ratio of 2–3. Long-term follow-up data were obtained from outpatient clinic, ambulatory electrocardiographic recordings and device interrogation reports. Follow-up for at least 1 year was available for 317 of 328 (96.6%) procedures, with a median follow-up duration of 5.4 years (interquartile range (IQR) = 4.1–6.3 years) for TC-SF and 2.3 years (IQR = 1.6–3.4 years) for TC-STSF.

### Statistical analysis

A propensity score was calculated for all eligible patients undergoing first-time RF ablation for VAs through binary logistic regression with ablation catheter (TC-STSF or TC-SF) as the binary outcome and baseline variables were used as covariates for estimating the propensity score. Propensity matching was performed in a 1:1 fashion using the nearest neighbour approach with a two-decimal calliper for age, sex, aetiology, LVEF and approach (endocardial or epicardial). If no match could be found, then the TC-STSF subject was removed from the analysis. Univariate analyses of dichotomous, categorical and continuous variables were performed to determine similarities or differences between TC-SF and TC-STSF in normal heart, ICM and NICM groups. Categorical data were compared using the Chi-square test. The distribution of continuous variables was assessed for normality with Shapiro-Wilk test. Student’s *t* test was used to compare normally distributed data and Mann-Whitney *U* test to compare non-parametric data between TC-SF and TC-STSF groups. Kaplan-Meier time event analysis was used to asses VA recurrence in each group, comparing TC-SF vs TC-STSF and significance determined by the log-rank test. Similarly, ICD shock recurrence and overall survival were compared in patients with ICM and NICM. Receiver operating characteristic (ROC) analysis was performed to find the average CF correlating with acute procedure success. Statistical analysis was performed using SPSS statistical software, version 26 (IBM Corp., Chicago, IL, USA).

## Results

### Patient details

Baseline characteristics are presented in Table 1. Procedures using either TC-SF or TC-STSF were well matched in patients’ gender distribution, age, distribution of cardiac aetiology and LVEF. There was no difference in the proportion of patients with implanted cardiac devices, indication for device implantation or indication for ablation.

**Table 1:** Baseline characteristics

	Normal heart (182)			ICM (76)			NICM (70)		
	TC-SF (91)	TC-STSF (91)	*P*	TC-SF (38)	TC-STSF (38)	*P*	TC-SF (35)	TC-STSF (35)	*P*
Male gender	40 (44)	41 (45)	0.9^a^	32 (84)	34 (90)	0.5^a^	27 (77)	25 (71)	0.09^a^
Age	50 ± 15	49 ± 16	0.7^b^	67.8 ± 8	68 ± 10	0.89^b^	57 ± 15	58 ± 15	0.7^b^
LVEF (%)	59 ± 3	59 ± 3	0.7^b^	39 ± 12	37 ± 12	0.4^b^	44 ± 13	42 ± 12	0.4^b^
Device									
ICD			1.0^a^	17(45)	17 (45)	1.0^a^	12 (34)	10 (29)	0.4^a^
CRT-D				9 (24)	10 (26)		6 (17)	11 (31)	
Pacemaker	0	1		1 (3)	1 (3)				
Device indication									
1ry prevention				13	11	0.45^a^	8	9	0.9^a^
2ry prevention				13	16		10	12	
Ablation indication									
Symptomatic	82	86	0.4^a^	13	12	0.5^a^	9	11	0.6^a^
ICD shocks				16	19		11	14	
VT storm				9	7		6	3	
Reduced LVEF	9	5					9	7	

Values are presented as count (percentage) or mean ± standard deviation

*1ry* primary, *2ry* secondary, *CRT-D* cardiac resynchronisation therapy-defibrillator, *CRT-P* cardiac resynchronisation therapy-pacing only, *ICD* implantable cardioverter defibrillator, *ICM* ischemic cardiomyopathy, *LVEF* left ventricular ejection fraction, *NICM* non-ischemic cardiomyopathy, *TC-SF* Thermocool Surround Flow catheter, *TC-STSF* Thermocool SmartTouch Surround Flow catheter, *VT* ventricular tachycardia

^a^Chi-square test

^b^Student’s *t* test

Pre-procedure cMRI was performed in 57/164 (35%) patients in the TC-SF group and 43/164 (26%) patients in the TC-STSF group (*p* = 0.09), with no difference in demographic characteristics between the patients in whom cMRI was performed in both groups. There was no difference between the two groups regarding the size of scar in patients with ICM, as measured by late gadolinium enhancement, TC-SF: median = 6 segments, IQR = 4–7; TC-STSF: median = 8 segments, IQR = 5–9, *p* = 0.12.

### Procedure characteristics

Procedure characteristics are shown in Table 2. There was no difference in the type of arrhythmia targeted, the approach used, procedure duration, duration of RF energy application or maximum power applied.

**Table 2:** Procedure details

	Normal heart (182)			ICM (76)			NICM (70)		
	TC-SF (91)	TC-STSF (91)	*P*	TC-SF (38)	TC-STSF (38)	*P*	TC-SF (35)	TC-STSF (35)	*P*
Type of VA									
VE	75	80	0.3^a^	10	10	1.0^a^	18	16	0.6^a^
VT	16	11		28	28		17	19	
Approach									
Endocardial	91	91		38	37	1.0^a^	34	34	1.0^a^
Epicardial-endocardial				0	1		1	1	
Procedure duration (min)	140 (120–170)	162 (135–186)	0.07^b^	245 (198–318)	210 (190–279)	0.3^b^	231 (162–253)	210 (139–301)	0.8^b^
Fluoroscopy time (min)	19 (14–30)	14 (8–25)	*0.04*^b^	33 (17–47)	27 (17–65)	0.2^b^	35 (15–47)	30 (20–39)	0.3^b^
RF time (min)	12 (7–23)	12 (6–23)	0.2^b^	27 (17–50)	27 (13–43)	0.98^b^	21 (12–36)	22 (19–32)	0.2^b^
Maximum power (W)	30 (27–35)	30 (25–30)	0.8^b^	35 (30–35)	30 (30–40)	0.7^b^	30 (30–35)	35 (30–40)	0.3^b^

Values are presented as count (percentage) or median (interquartile range)

*ICM* ischemic cardiomyopathy, *min* minute, *NICM* non-ischemic cardiomyopathy, *RF* radiofrequency, *TC-SF* Thermocool Surround Flow catheter, *TC-STSF* Thermocool SmartTouch Surround Flow catheter, *VA* ventricular arrhythmia, *VE* ventricular ectopics, *VT* ventricular tachycardia, *W* Watts

^a^Chi-square test

^b^Mann-Whitney test

In patients presenting with VT, there was no difference in the number of VTs induced between both study groups (TC-SF: median = 1, IQR = 1–2; TC-STSF: median = 1, IQR = 1–2; *p* = 0.7). The clinical VT as well as any induced sustained VT was targeted for ablation.

With all etiologies combined, fluoroscopy time was shorter with TC-STSF (median: 16 min, IQR: 9–27) than TC-SF (median: 21 min, IQR: 13–31), *p* = 0.008. However, this difference was only significant in the normal heart group (TC-SF: median = 19 min, IQR = 14–30, TC-STSF: median = 14 min, IQR = 8–25, *p* = 0.04) but not in ICM or NICM groups (Table 2).

When examining patients undergoing VE and VT ablation separately (Table 3), fluoroscopy time was shorter with the use of TC-STSF in VE ablation (TC-SF: median = 23 min, IQR = 15–41, TC-STSF: median = 17 min, IQR = 8–25, *p* = 0.009). There were no other differences in procedure duration, duration of RF energy application or maximum power applied.

**Table 3:** Comparison between the two catheters based on type of ventricular arrhythmia

	Ventricular ectopy			Ventricular tachycardia		
	TC-SF (103)	TC-STSF (106)	*P*	TC-SF (61)	TC-STSF (58)	*P*
Procedure duration (min)	165 (140–253)	168 (133–208)	0.6^a^	199 (146–248)	210 (176–292)	0.4^a^
Fluoroscopy time (min)	23 (15–41)	17 (8–25)	*0.009*^a^	24 (19–31)	28 (15–37)	0.8^a^
RF time (min)	14 (8–20)	13 (7–22)	0.3^a^	23 (12–30)	28 (19–45)	0.4^a^
Maximum power (W)	30 (30–35)	30 (27–30)	0.9^a^	35 (30–35)	35 (30–40)	0.9^a^
Acute success	85 (83)	95 (90)	0.14^b^	49 (80)	46 (79)	0.9^b^
30-day complications	6 (6)	4 (4)	0.5^b^	5 (8)	6 (10)	0.3^b^
Tamponade	4	2		0	3	
Stroke	1	0				
Heart block				0	1	
Vascular	1	0		2	2	
Death				1	1	
Pulmonary embolism				1	0	
Pneumonia				1	0	
Pericarditis	0	1				
Coronary dissection	0	1				

Values are presented as median (interquartile range) or count (percentage)

*Min* minute, *RF* radiofrequency, *TC-SF* Thermocool Surround Flow catheter, *TC-STSF* Thermocool SmartTouch Surround Flow catheter, *W* Watts

^a^Mann-Whitney test

^b^Chi-square test

In the TC-STSF group, the median for average CF during RF delivery was 13 g, IQR = 10–19 and the median for maximum CF = 64 g, IQR = 42–80. The average CF in patients undergoing VA ablation for non-outflow LV sites was higher with the trans-mitral approach (median = 19 g, IQR = 12–25) than with the trans-aortic approach (median = 14 g, IQR = 9–18, *p* = 0.02). Likewise, the trans-mitral approach was associated with higher maximum CF (median = 90 g, IQR = 69–103) than the trans-aortic approach (median = 69, IQR = 41–88, *p* = 0.03).

An average CF greater than 5 g during RF delivery could not be reached in 4 of 164 patients (2.4%); 2 cases involved ablation in dilated RV outflow tracts, one involved the anterior mitral annulus and one involved basal LV ablation for LV summit VE.

### Acute success and 30-day complications

There was no difference between procedures utilising either TC-SF or TC-STSF in acute success (TC-SF: 134/164 (82%), TC-STSF: 141/164 (86%), *p* = 0.3). No difference in acute success was observed between the two catheters when VE and VT ablations were analysed separately (Table 3) or in normal hearts, ICM or NICM (Table 4).

**Table 4:** Acute success and complications

	Normal heart (182)			ICM (76)			NICM (70)		
	TC-SF (91)	TC-STSF (91)	*P*	TC-SF (38)	TC-STSF (38)	*P*	TC-SF (35)	TC-STSF (35)	*P*
Acute success	76 (84)	83 (91)	0.1^a^	33 (87)	36 (95)	0.2^a^	25 (71)	22 (63)	0.4^a^
30-day complications	4 (4)	3 (3)	0.5^a^	5 (13)	4 (11)	0.6^a^	2 (6)	4 (11)	0.7^a^
Tamponade	2	1		1	2		1	2	
Stroke	1	0							
Heart block				0	1				
Death				1	1				
Pneumonia				1	0				
Pulmonary embolism				1	0				
Vascular	1	0		1	0		1	2	
Pericarditis	0	1							
Coronary dissection	0	1							

Values are presented as count (percentage)

*ICM* ischemic cardiomyopathy, *NICM* non-ischemic cardiomyopathy, *TC-SF* Thermocool Surround Flow catheter, *TC-STSF* Thermocool SmartTouch Surround Flow catheter

^a^Chi-square test

Likewise, there was no difference in complications between TC-SF (11 of 164, 6.7%) and TC-STSF (11 of 164, 6.7%, *p* = 1.0) catheter groups, and this applied to VE and VT ablations (Table 3) as well as normal hearts, ICM and NICM (Table 4). There was no association between maximum CF and incidence of tamponade, but the numbers were too small to demonstrate a difference. There were two deaths within 30 days of the procedure; both patients had advanced heart failure with severely depressed LVEF (< 20%).

Tamponade requiring drainage occurred in 9 (2.7%) procedures, 8 of which resolved with pericardiocentesis and one associated with TC-STSF use required surgical repair. Of the four tamponades occurring with TC-SF, two occurred with ablation in the right ventricular outflow tract (RVOT), one with ablation in the right coronary cusp and one occurred 1.5 months later due to pericarditis following an epicardial ablation. Of the five tamponades occurring with TC-STSF, two occurred with ablation in the left ventricle, one with ablation in the aorto-mitral continuity, one with ablation in the RVOT and one with ablation in the RV free wall.

One patient sustained acute left main coronary injury successfully stented emergently, due to manipulation of TC-STSF in the aortic root to target a papillary muscle VE, one case of complete heart block occurred, and one patient suffered a minor stroke. No other thromboembolic events were observed.

### Long-term follow-up

**Fig. 2:**
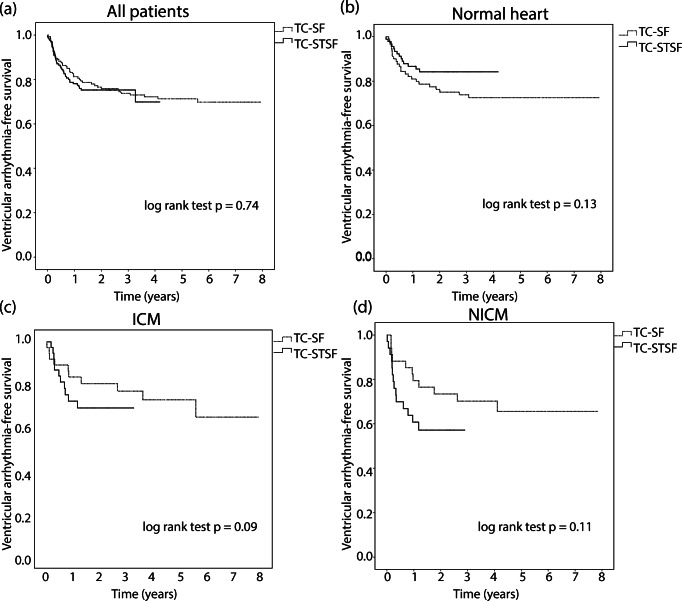
Kaplan-Meier survival analysis of ventricular arrhythmia-free survival comparing the TC-SF (dotted line) and TC-STSF (solid line) groups in the whole study population (**a**), in normal hearts (**b**), ischemic cardiomyopathy (**c**) and non-ischemic cardiomyopathy (**d**). ICM = ischemic cardiomyopathy; NICM = non-ischemic cardiomyopathy; TC-SF = Thermocool Surround Flow; TC-STSF = Thermocool SmartTouch Surround Flow

**Fig. 3:**
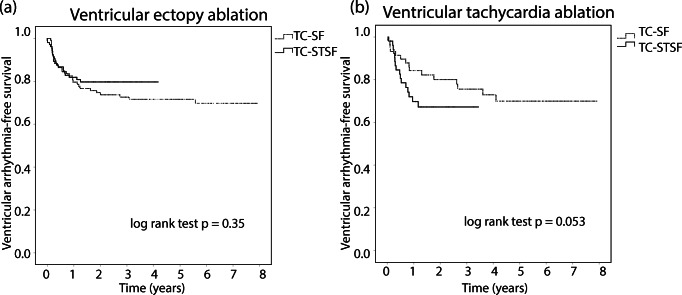
Kaplan-Meier survival analysis of ventricular arrhythmia-free survival comparing the TC-SF (dotted line) and TC-STSF (solid line) groups in patients undergoing VE ablation (**a**) and in patients undergoing VT ablation (**b**). TC-SF = Thermocool Surround Flow; TC-STSF = Thermocool SmartTouch Surround Flow; VE = ventricular ectopics; VT = ventricular tachycardia

When comparing TC-SF and TC-STSF catheter groups, there was no difference in VA-free survival in the whole study group (TC-SF: mean estimated VA-free survival time = 5.9 years, 95% CI = 5.4–6.4, TC-STSF: mean = 3.2 years, 95% CI = 3–3.5, log-rank *p* = 0.74, Fig. 2a), normal hearts (TC-SF: mean estimated VA-free survival time = 6 years, 95% CI = 5.3–6.7, TC-STSF: mean = 3.6 years, 95% CI = 3.3–3.9, log-rank *p* = 0.13, Fig. 2b), ICM (TC-SF: mean estimated VA-free survival time = 5.9 years, 95% CI = 4.9–7, TC-STSF: mean = 2.4 years, 95%, CI = 2–2.9, log-rank *p* = 0.15, Fig. 2c) or NICM (TC-SF: mean estimated VA-free survival time = 5.6 years, 95% CI = 4.5–6.7, TC-STSF: mean = 1.8 years, 95% CI = 1.4–2.3, log-rank *p* = 0.11, Fig. 2d). No difference in VA-free survival was observed between the two catheters when comparing patients who underwent VE ablation (TC-SF: mean estimated VA-free survival time = 5.9 years, 95% CI = 5.3–6.5, TC-STSF: mean = 3.4 years, 95% CI = 3.1–3.7, log-rank *p* = 0.35, Fig. 3a) or VT ablation (TC-SF: mean estimated VA-free survival time = 6 years, 95% CI = 5.2–6.9, TC-STSF: mean = 2.4 years, 95% CI = 2.0–2.8, log-rank *p* = 0.053, Fig. 3b).

**Fig. 4:**
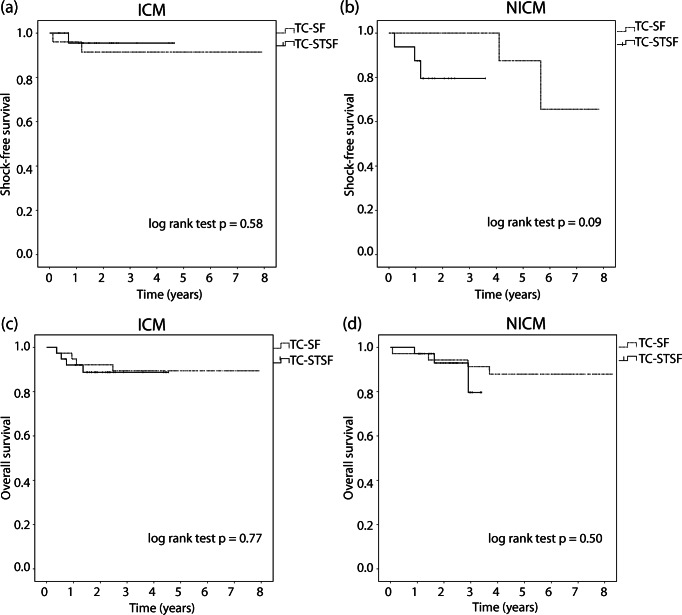
**a**, **b** Kaplan-Meier survival analysis of shock-free survival comparing the TC-SF (dotted line) and TC-STSF (solid line) groups in patients with ischemic cardiomyopathy (**a**) and with non-ischemic cardiomyopathy (**b**). **c**, **d** Kaplan-Meier survival analysis of overall survival comparing the TC-SF (dotted line) and TC-STSF (solid line) groups in patients with ischemic cardiomyopathy (**c**) and with non-ischemic cardiomyopathy (**d**). ICM = ischemic cardiomyopathy; NICM = non-ischemic cardiomyopathy; TC-SF = Thermocool Surround Flow; TC-STSF = Thermocool SmartTouch Surround Flow

There was no difference in shock-free survival in either patients with ICM (TC-SF: mean estimated shock-free survival time = 7.3 years, 95% CI = 6.5–8.1, TC-STSF: mean = 4.5 years, 95% CI = 4.1–4.8, log-rank *p* = 0.58, Fig. 4a) or NICM (TC-SF: mean estimated shock-free survival time = 6.9 years, 95% CI = 5.8–8, TC-STSF: mean = 3 years, 95% CI = 2.4–3.6, log-rank *p* = 0.09, Fig. 4b). Likewise, there was no difference in overall survival in either patients with ICM (TC-SF: mean estimated survival time = 7.2 years, 95% CI = 6.6–7.9, TC-STSF: mean = 4.1 years, 95% CI = 3.7–4.5, log-rank *p* = 0.77, Fig. 4c) or NICM (TC-SF: mean estimated survival time = 7.6 years, 95% CI = 6.9–8.3, TC-STSF: mean = 3.2 years, 95% CI = 3–3.4, log-rank *p* = 0.50, Fig. 4d).

In patients who had undergone VT ablation, there was no difference in the utilisation of antiarrhythmic medications during long-term follow-up (Table 5).

**Table 5:** Antiarrhythmic medications during follow-up in patients who underwent ablation for ventricular tachycardia

	TC-SF *N* = 61	TC-STSF *N* = 58	*P* value^a^
Beta blockers	40 (66)	37 (64)	0.8
Amiodarone	23 (38)	19 (33)	0.3
Sotalol	6 (10)	7 (12)	0.2
Mexiletine	6 (10)	3 (5)	0.3

Values are presented as count (percentage)

*TC-SF* Thermocool Surround Flow, *TC-STSF* Thermocool SmartTouch Surround Flow

^a^Chi-square test

### Redo of TC-SF procedures with TC-STSF

Of the 164 patients in the TC-SF catheter group, 7 patients subsequently underwent repeat ablation using the TC-STSF catheter for recurrent VA. Reasons for failure in the index procedure were adjudicated by 2 senior electrophysiologists. In two cases, recurrence of VT occurred after endocardial ablation of NICM requiring an endocardial/epicardial approach. Difficulties with mapping were identified in three cases and non-inducibility in one case. Recurrence due to mid-myocardial location of substrate occurred in one case. In two cases, the use of CF was considered to aid the success of the subsequent procedure for VEs mapped to the superior RV inflow in one and the aorto-mitral continuity in another.

## Discussion

This study is the largest propensity-matched series assessing the role of CF in the ablation of VAs to date and the first to investigate whether measuring CF improves on the outcomes achieved by entire tip irrigation. The main finding from this study is that the addition of CF sensing to a 56-hole irrigation RF ablation catheter was not associated with improved acute procedural success, complications or long-term outcomes. Prior studies in sheep models and small human series suggested that measuring CF improved substrate characterisation and lesion formation, especially in scar zones [3, 4]. CF achieved in different regions of the left ventricle has been found to depend on the approach used (whether transseptal or transaortic) [5]. The consistent use of both approaches to the left ventricle for patients with scar-dependent VA at our center might have lessened the effect of measuring CF. Although the size of our study is insufficient to make firm conclusions, we postulate that the use of CF may aid ablation in specific areas within the ventricles. Areas such as the inflow portion of the right ventricle, the aorto-mitral continuity, the subaortic vestibule (infero-septal recess), particularly when the ascending aorta is horizontally situated or tortuosity of the descending aorta is encountered, may benefit from the use of CF. In these situations, the manual feedback afforded to the operator is reduced and often requires significant catheter torque to manoeuvre the catheter. Optimising catheter-tissue contact may also be of greater value with deeper intramyocardial foci. Additionally, CF sensing catheters enable the operator to visualise the direction of force which may be advantageous during epicardial ablation. Our propensity-matched first-time cases included only three epicardial procedures, which were insufficient to demonstrate such an advantage.

The failure of CF sensing to offer increased efficacy in our series may be explained by the fact that there is no clear guidance on how to act on CF values in different ventricular sites of varying wall thickness. The use of surrogate markers of effective RF lesion formation, such as initial impedance fall, shown to be a correlate of good CF [6], and electrical unexcitability of ablated tissue, may have limited the relative utility of CF sensing. Other variables which influence final lesion formation are not accounted for by the CF value displayed, such as the duration of RF energy application, catheter tip angulation as well as the spatio-temporal variation in contact caused by cardiac and respiratory motion [7, 8]. These might in the future be encompassed by a derived, weighted parameter combining power, time and CF, as recent reports suggest [9]. Due to the heterogeneity in tissue thickness and composition in both ventricles as well as in diverse cardiac pathologies, region- and tissue-specific parameters will be required to maximise effective lesion formation without compromising safety.

Our data shows both the TC-SF and TC-STSF catheters to be equally safe in treating VAs. This is reassuring as prior concerns were expressed regarding the safety of the TC-SF design due to a higher reported frequency of cardiac perforation as well as atrio-esophageal fistula in left atrial ablation procedures. This was corroborated by increased steam pops in canine models and in vitro simulations [10, 11], prompting the manufacturer to issue a safety notice for the TC-SF catheter in 2014. The safety advantage gained by adding CF sensing to preexisting platforms may be offset by an increase in catheter stiffness, as CF-sensing catheters were associated with higher reported rates of atrio-esophageal fistula as adverse events in left atrial procedures [12]. This is concordant with our observation of a higher number of adverse events related to catheter manipulation with TC-STSF, including cardiac perforation and coronary injury due to catheter manipulation in the aortic root. This, although not statistically significant, would warrant caution while using TC-STSF in more vulnerable locations, e.g. RVOT and aortic root. CF is monitored by operators during ablation, but abrupt catheter movements during manipulation can lead to unanticipated and potentially injurious rises in CF.

Compared with the TC-SF catheter, the use of TC-STSF was associated with reduced fluoroscopy time, mainly in normal heart VA ablation. A similar observation was noted in a previous comparison of both catheters, which mainly comprised atrial ablation procedures [13]. This may indicate that operator confidence in catheter tip positioning and contact is less reliant on fluoroscopy when CF data is available especially in the outflow tracts. An alternative explanation might be that the TC-STSF cases were more recent and there has been a steady decline in fluoroscopy exposure over the past 10 years because of other factors, e.g. improvement in mapping systems. However, our finding that fluoroscopy times did not decrease with structural heart disease in the same epoch would argue against this.

Concordant with our findings, a previous observational study investigating the utility of CF in VAs using six-hole irrigation systems failed to show an improvement in procedure outcome or safety profile [14]. The findings are also analogous to the literature available on CF sensing in atrial ablation procedures. While non-randomised studies showed lower CF values to be associated with pulmonary vein reconnection [15, 16], randomised controlled trials failed to demonstrate that the use of CF improves intermediate to long-term outcome [17].

### Limitations

This was a retrospective single-center study. Specific parameter settings were left to operator discretion. However, case-specific ablation endpoints were the same for both catheters. The large number of cases and the diversity of etiologies provide real-world experience of the use of both catheters.

The catheters were used mostly sequentially. Due to the lack of propensity-matched cases and as only first-time ablations were included, the study comprised a small proportion of cases using the epicardial approach and no surgical ablation cases. Image integration with pre-procedure cMRI was not available; it would have been useful to compare the area of scar as measured by cMRI and by voltage mapping both with and without CF data. As the data was collected retrospectively, direct comparison between contact force achieved in the left ventricle with transmitral and transaortic approach at the same sites in the left ventricle was not possible.

The modest number of patients undergoing VT ablation may have been insufficient to find a small potential difference in outcomes due to the use of contact force in this subset.

## Conclusion

Both TC-SF and TC-STSF catheters are safe and effective in treating VA. The addition of CF sensing to a 56-hole open-irrigation RF catheter did not confer additional benefits in terms of safety, acute success or long-term outcomes in ablating VA, but reduced fluoroscopy time in normal heart VA.

## References

[1] Mallidi J, Nadkarni GN, Berger RD, Calkins H, Nazarian S (2011). Meta-analysis of catheter ablation as an adjunct to medical therapy for treatment of ventricular tachycardia in patients with structural heart disease. Heart Rhythm.

[2] Jaïs P, Maury P, Khairy P, Sacher F, Nault I, Komatsu Y, Hocini M, Forclaz A, Jadidi AS, Weerasooryia R, Shah A, Derval N, Cochet H, Knecht S, Miyazaki S, Linton N, Rivard L, Wright M, Wilton SB, Scherr D, Pascale P, Roten L, Pederson M, Bordachar P, Laurent F, Kim SJ, Ritter P, Clementy J, Haïssaguerre M (2012). Elimination of local abnormal ventricular activities: a new end point for substrate modification in patients with scar-related ventricular tachycardia. Circulation..

[3] Sacher F, Wright M, Derval N, Denis A, Ramoul K, Roten L, Pascale P, Bordachar P, Ritter P, Hocini M, Dos Santos P, Haissaguerre M, Jais P (2013). Endocardial versus epicardial ventricular radiofrequency ablation utility of in vivo contact force assessment. Circ Arrhythm Electrophysiol.

[4] Elsokkari I, Sapp JL, Doucette S, Parkash R, Gray CJ, Gardner MJ, Macintyre C, AbdelWahab A (2018). Role of contact force in ischemic scar-related ventricular tachycardia ablation; optimal force required and impact of left ventricular access route. J Interv Card Electrophysiol.

[5] Jesel L, Sacher F, Komatsu Y (2014). Characterization of contact force during endocardial and epicardial ventricular mapping. Circ Arrhythm Electrophysiol.

[6] Reichlin T, Knecht S, Lane C (2014). Initial impedance decrease as an indicator of good catheter contact: insights from radiofrequency ablation with force sensing catheters. Heart Rhythm.

[7] Gallagher N, Fear EC, Byrd IA, Vigmond EJ (2013). Contact geometry affects lesion formation in radio-frequency cardiac catheter ablation. PLoS One.

[8] Sarkozy A, Shah D, Saenen J (2015). Contact force in atrial fibrillation: role of atrial rhythm and ventricular contractions: co-force atrial fibrillation study. Circ Arrhythm Electrophysiol.

[9] Casella M, Gasperetti A, Gianni C (2019). Ablation index as a predictor of long-term efficacy in premature ventricular complex ablation: a regional target value analysis. Heart Rhythm.

[10] Guerra JM, Jorge E, Raga S, Gálvez-Montón C, Alonso-Martín C, Rodríguez-Font E, Cinca J, Viñolas X (2013). Effects of open-irrigated radiofrequency ablation catheter design on lesion formation and complications: in vitro comparison of 6 different devices. J Cardiovasc Electrophysiol.

[11] Winterfield JR, Jensen J, Gilbert T, Marchlinski F, Natale A, Packer D, Reddy V, Mahapatra S, Wilber DJ (2016). Lesion size and safety comparison between the novel flex tip on the FlexAbility ablation catheter and the solid tips on the thermo cool and thermo cool SFl. J Cardiovasc Electrophysiol.

[12] Black-Maier E, Pokorney SD, Barnett AS (2017). Risk of atrioesophageal fistula formation with contact force–sensing catheters. Heart Rhythm.

[13] Gonna H, Domenichini G, Zuberi Z, Norman M, Kaba R, Grimster A, Gallagher MM (2017). Initial clinical results with the ThermoCool® SmartTouch® surround flow catheter. Europace..

[14] Hendriks AA, Akca F, Dabiri Abkenari L, Khan M, Bhagwandien R, Yap SC, Wijchers S, Szili-Torok T (2015). Safety and clinical outcome of catheter ablation of ventricular arrhythmias using contact force sensing: consecutive case series. J Cardiovasc Electrophysiol.

[15] Kuck KH, Reddy VY, Schmidt B (2012). A novel radiofrequency ablation catheter using contact force sensing: toccata study. Heart Rhythm.

[16] Neuzil P, Reddy VY, Kautzner J (2013). Electrical reconnection after pulmonary vein isolation is contingent on contact force during initial treatment: results from the EFFICAS i study. Circ Arrhythm Electrophysiol.

[17] Reddy VY, Dukkipati SR, Neuzil P, Natale A, Albenque JP, Kautzner J, Shah D, Michaud G, Wharton M, Harari D, Mahapatra S, Lambert H, Mansour M (2015). Randomized, controlled trial of the safety and effectiveness of a contact force-sensing irrigated catheter for ablation of paroxysmal atrial fibrillation: results of the TactiCath contact force ablation catheter study for atrial fibrillation (TOCCASTAR) S. Circulation..

